# Erythema nodosum in northern Finland between 1996 and 2019: A register‐based study

**DOI:** 10.1111/1346-8138.17264

**Published:** 2024-05-06

**Authors:** Riina Lantto, Jari Jokelainen, Laura Huilaja, Suvi‐Päivikki Sinikumpu

**Affiliations:** ^1^ Faculty of Medicine University of Oulu Oulu Finland; ^2^ Northern Finland Birth Cohorts, Arctic Biobank, and Infrastructure for Population Studies, Faculty of Medicine University of Oulu Oulu Finland; ^3^ Department of Dermatology, Oulu University Hospital, and Research Unit of Clinical Medicine University of Oulu Oulu Finland

**Keywords:** characteristics, erythema nodosum, etiology, symptoms

## Abstract

Erythema nodosum (EN) is seen at any age with varying and often unidentified etiology. We studied the etiology and characteristics of EN in Northern Finland. Medical records of all patients with a diagnosis code for EN between 1996 and 2019 from Oulu University Hospital were retrieved and analyzed. There were in total 142 EN cases with a female predominance (*n* = 112, 72.9%). The mean age of the patients was 35.9 years. There were five cases diagnosed with EN in those younger than 2 years of age. Almost one third had EN nodules in multiple anatomical locations. In addition to skin findings, systemic symptoms were common (81.0%), and seen more often in men (*p* < 0.05). In children and adolescents, the most common etiological factors were gastroenteritis caused by ‘Yersinia, Salmonella or Campylobacter’, followed by inflammatory bowel diseases and hormonal contraception. Bacterial infections were the most common etiological factor among adults. In 28.2% of the cases there was no identified causative factor. In this study, EN was seen surprisingly often in small children. Etiological factors varied markedly among different age groups and symptoms differed between the sexes in adults. These aspects should be taken into account when diagnosing EN patients.

## 1 INTRODUCTION

Panniculitis is a condition where the subcutaneous fat tissue becomes inflamed. The most common clinical form of panniculitis is erythema nodosum (EN), characterized by painful, red, rounded nodules traditionally localized on the anterior surface of the limbs. The most common localization is the shins, although other localizations are possible.^1^ The etiology of EN is still unknown with around half of the cases considered idiopathic.^1^, ^2^, ^3^ The known causes for EN include infections, sarcoidosis, pregnancy, inflammatory bowel diseases (IBD), drugs (such as contraceptive pills), and neoplastic diseases.^1^, ^2^, ^3^, ^4^ The current annual incidence of EN is estimated to be around 15 per 100 000 persons, varying depending on the incidence rate of the causal conditions between populations.^2^ EN is more common among females, appearing up to five times more often in women than in men.^2^, ^3^ EN is most often diagnosed in patients from their teens to their 30s, but it can be seen at any age.^2^ The objective of this study was to analyze the characteristics and etiology associated with EN in patients in the Oulu University Hospital (OUH) in Northern Finland.

## 2 MATERIALS AND METHODS

The International Classification of Diseases (ICD)‐10 code for EN (L52) was used for retrieving patient records from the OUH database. Every patient that had either visited an outpatient clinic or been admitted to OUH with the aforementioned diagnosis between January 1, 1996 and December 31, 2019 was included in this study. The authors reviewed all patient records manually and those with an actual diagnosis other than EN were not included to the study (exclusions included miscoding and those cases in which diagnoses were further confirmed to be other than EN, i.e. other panniculitis). OUH belongs to the Northern Ostrobothnia Hospital District (NOHD) that provides special healthcare for a Finnish population of 415 867.

## 3 RESULTS

A data query returned 170 cases with an L52 code. When the uncertain cases were excluded, there was a total of 142 cases of EN included in this study with a female predominance (*n* = 112; 78.9%). Mean age at the time of EN diagnosis was 35.9 years (median 33.0 [range, 1–82 years]) and 11.8 years (median 14.0 [range, 1–19 years]) among children and adolescents. There were five cases diagnosed with EN in those younger than 2 years of age. Most commonly, the diagnosis of EN was made at the department of internal medicine (*n* = 61, 43.0%) followed by the dermatology department (*n* = 50, 35.2%). In children or adolescents, the diagnosis was most often given at the pediatric clinic (*n* = 17, 58.6%). (Tables 1 and 2).

**TABLE 1:** Baseline characteristics of patients diagnosed with erythema nodosum.

	Men	Women	Total	*p*
Total number of patients, *n* (%)	30 (21.1)	112 (78.9)	142	
Age, mean (SD), years	29.6 (17.9)	37.5 (19.3)	35.9 (19.2)	0.071
Age group, *n* (%) years				0.172
0–19	10 (33.3)	19 (17.0)	29 (20.4)	
20–39	12 (40.0)	49 (43.8)	61 (43.0)	
40–64	7 (23.3)	31 (27.7)	38 (26.8)	
≥ 65	1 (3.3)	13 (11.6)	14 (9.9)	
Smoking status, *n* (%)				0.791
Smoker	4 (13.3)	17 (15.2)	21 (14.8)	
Non‐smoker	12 (40.0)	35 (31.2)	47 (33.1)	
Ex‐smoker	2 (6.7)	12 (10.7)	14 (9.9)	
No information	12 (40.0)	48 (42.9)	60 (42.3)	
Number of nodules, *n* (%)				0.649
Single	1 (3.3)	6 (5.4)	7 (4.9)	
Multiple	29 (96.7)	106 (94.6)	135 (95.1)	
Anatomical site of nodules, *n* (%)				0.971
Lower limb	2 (6.7)	8 (7.1)	10 (7.0)	
Both lower limbs	20 (66.7)	72 (64.3)	92 (64.8)	
Multiple areas^a^	8 (26.7)	32 (28.6)	40 (28.2)	
Ulceration of nodules, *n* (%)	1 (3.3)	3 (2.8)	4 (2.9)	0.866
Systemic symptoms, *n* (%)	29 (96.7)	86 (76.8)	115 (81.0)	0.014
Malaise	4 (13.3)	14 (12.5)	18 (12.7)	0.903
Fever	21 (70.0)	44 (39.3)	65 (45.8)	0.003
Nausea	0 (0.0)	3 (2.7)	3 (2.1)	0.365
Arthralgia	11 (36.7)	50 (44.6)	61 (43.0)	0.433
Symptoms of respiratory infection	13 (43.3)	35 (31.2)	48 (33.8)	0.214
Swollen ankles	13 (43.3)	33 (29.5)	46 (32.4)	0.149
Etiology, *n* (%)				
Medication^b^				
Hormonal contraception	0 (0.0)	18 (16.1)	18 (12.7)	NF
Penicillin	1 (3.3)	4 (3.6)	5 (3.5)	0.950
Sulfonamides	0 (0.0)	1 (0.9)	1 (0.7)	0.603
TNF‐alpha inhibitors	1 (3.3)	1 (0.9)	2 (1.4)	0.314
Infection	13 (43.3)	52 (46.4)	65 (45.8)	0.762
Bacterial^c^	10 (33.3)	48 (42.9)	58 (40.8)	0.346
*Streptococcal*	1 (3.3)	11 (9.8)	12 (8.5)	0.257
*Yersinia, Salmonella, Campylobacter*	6 (20.0)	6 (5.4)	12 (8.5)	0.010
*Mycoplasma*	0 (0.0)	1 (0.9)	1 (0.7)	0.603
*Tularemia*	0 (0.0)	4 (3.6)	4 (2.8)	0.294
*Chlamydia trachomatis*	0 (0.0)	1 (0.9)	1 (0.7)	0.603
Viral infection	2 (6.7)	4 (3.6)	6 (4.2)	0.454
Fungal infection	0 (0.0)	1 (0.9)	1 (0.7)	0.603
Other causes				
Sarcoidosis	4 (13.3)	13 (11.6)	17 (12.0)	0.971
Pregnancy	0 (0.0)	5 (4.5)	5 (4.5)	NF
Inflammatory bowel disease	4 (13.3)	6 (5.4)	10 (7.0)	0.265
Malignancy	1 (3.3)	3 (2.7)	4 (2.8)	0.847
Behcet's disease	0 (0.0)	1 (5.3)	1 (4.3)	NF
No known etiology	8 (26.7)	32 (28.6)	40 (28.2)	0.837

*Note*: Data are presented as means, standard deviation (SD) and range, and as proportions for categorical variables. A Chi square test or Fisher's exact test were used to test differences between sexes and between age groups. All statistical analyses were performed using R (version 4.3.0, R Foundation for Statistical Computing, Vienna, Austria) and a *p*‐value of 0.05 was considered statistically significant.

Abbreviation: NF, Not feasible; TNF, tumor necrosis factor.

^a^ Including cases with symptoms in one/both lower extremities and ≥ one other area.

^b^ As a suspected etiology of erythema nodosum.

^c^ Any bacterial infection. There was a suspicion of several infections in one woman.

**TABLE 2:** Characteristics of patients with erythema nodosum by age groups.

Age‐group by years	0–19	20–39	40–64	≥65	*p*
Total number of patients, *n*	29	61	38	14	
Age, mean (SD), years	11.8 (6.2)	29.5 (5.8)	51.1 (7.2)	72.2 (5.1)	
Sex, *n* (%)					0.172
Female	19 (65.5)	49 (80.3)	31 (81.6)	13 (92.9)	
Number of nodules, *n* (%)					0.159
Single	0 (0.0)	2 (3.3)	3 (7.9)	2 (14.3)	
Multiple	29 (100)	59 (96.7)	35 (92.1)	12 (85.7)	
Anatomical site of nodules, *n* (%)					0.025
Lower limb	0 (0.0)	2 (3.3)	5 (13.2)	3 (21.4)	
Both lower limbs	20 (69.0)	46 (75.4)	19 (50.0)	7 (50.0)	
Multiple areas^a^	9 (31.0)	13 (21.3)	14 (36.8)	4 (28.6)	
Ulceration of nodules, *n* (%)	0 (0.0)	2 (3.4)	1 (2.7)	1 (7.1)	0.608
Systemic symptoms, *n* (%)	24 (82.8)	53 (86.9)	30 (78.9)	8 (57.1)	0.082
Malaise	6 (20.7)	7 (11.5)	4 (10.5)	1 (7.1)	0.511
Fever	17 (58.6)	26 (42.6)	18 (47.4)	4 (28.6)	0.275
Nausea	0 (0.0)	2 (3.3)	1 (2.6)	0 (0.0)	0.711
Arthralgia	7 (24.1)	29 (47.5)	23 (60.5)	2 (14.3)	0.003
Respiratory infection symptoms	14 (48.3)	21 (34.4)	12 (31.6)	1 (7.1)	0.064
Swollen ankles	8 (27.6)	23 (37.7)	12 (31.6)	3 (21.4)	0.599
Etiology, *n* (%)					
Infection	18 (62.1)	28 (45.9)	15 (39.5)	4 (28.6)	0.146
Bacterial^b^	14 (48.3)	26 (42.6)	14 (36.8)	4 (28.6)	0.600
*Streptococcal infection*	4 (13.8)	5 (8.2)	2 (5.3)	1 (7.1)	0.658
*Yersinia, Salmonella, Campylobacter*	6 (20.7)	2 (3.3)	4 (10.5)	0 (0.0)	0.026
Viral infection	3 (10.3)	3 (4.9)	0 (0.0)	0 (0.0)	0.168
Fungal infection	0 (0.0)	1 (1.6)	0 (0.0)	0 (0.0)	0.720
Medication^c^					
Hormonal contraception	4 (13.8)	13 (21.3)	1 (2.6)	0 (0.0)	0.022
Penicillin	0 (0.0)	4 (6.6)	1 (2.6)	0 (0.0)	0.346
Sulfonamides	0 (0.0)	0 (0.0)	1 (2.6)	0 (0.0)	0.431
TNF‐alpha inhibitors	1 (3.4)	0 (0.0)	1 (2.6)	0 (0.0)	0.503
Other causes					
Inflammatory bowel disease	4 (13.8)	3 (4.9)	3 (7.9)	0 (0.0)	0.378
Malignancy	0 (0.0)	0 (0.0)	3 (7.9)	1 (7.1)	0.067
Sarcoidosis	0 (0.0)	7 (11.5)	8 (21.1)	2 (14.3)	0.038
Pregnancy	0 (0.0)	5 (8.2)	0 (0.0)	0 (0.0)	NF
Behcet's disease	0 (0.0)	0 (0.0)	1 (2.6)	0 (0.0)	NF

*Note*: Data are presented as means, standard deviation (SD) and range, and as proportions for categorical variables. A Chi^2^ test or Fischer's exact test were used to test differences between sexes and between age groups. All statistical analyses were performed using R (version 4.3.0, R Foundation for Statistical Computing, Vienna, Austria) and a *p*‐value of 0.05 was considered statistically significant.

Abbreviation: NF, Not feasible; TNF, tumor necrosis factor.

^a^ Including cases with symptoms in one/both lower extremities and ≥ one other area.

^b^ As a suspected etiology of erythema nodosum.

^c^ Any bacterial infection. There was a case with suspicion of several infections among women.

The occurrence of systemic symptoms was more common in men than in women (*p* = 0.014). Fever was the most common systemic symptom in men (*p* < 0.01), whereas it was arthralgia among women. (Table 1). The occurrence of joint pain was most common in people aged 40–64 years, and least common in people over 65‐year‐olds (*p* < 0.01) (Table 2).

As an etiological factor, gastroenteritis caused by ‘Yersinia, Salmonella or Campylobacter’ was found more often in males than in females (*p* = 0.01) (Table 1), and more often in the youngest age groups compared to the older ones (*p* = 0.026) (Table 2). Malignancy was found in four cases (2.8%): three were internal carcinomas and one leukemia; all the patients were ≥40 years.

In 18.3% (*n* = 26) of cases a skin biopsy was taken to confirm the diagnosis, most commonly among those ≥40 years (*p* < 0.01). At the time of diagnosing EN, a chest X‐ray was performed in most cases (89.4%), but least often in the youngest age group (*p* = 0.039). The erythrocyte sedimentation rate was analyzed in 127/142 (89.4%) of the cases, the mean (SD, min‐max) value being 39.3 mm/h (28.9, 2.0–104.0) in children (normal range, 1–15 mm/h) and 44.4 mm/h (28.0, 2.0–107.0) in adults (normal range, varying from <10 to <45 depending on age and sex).

## 4 DISCUSSION

This study assessed the etiology and clinical characteristics in 142 patients with EN using OUH hospital records. Surprisingly, many children or adolescents with EN were seen in our study population (*n* = 29). EN is reported to be quite uncommon among children, and especially rare among those under 2 years.^5^ However, there were five cases diagnosed with EN aged ≤2 years in our study. In line with previous studies,^3^ females comprised the majority (78.9%) of our cases. Nevertheless, among children, especially in those under 12 years, no sex prevalence has been reported.^4^, ^5^ In our study, there was a predominance of females, also in the youngest age group (0–19 years).

The etiology of EN varies depending on geographical area, sex and age.^1^, ^3^, ^5^ However, detailed data about etiological factors are scarce. In our study, infections, especially bacterial infections, were the most common known etiology. Oral contraceptives, IBD, and sarcoidosis were also notable factors. In contrast to many other studies,^3^, ^4^, ^6^ there were no cases with tuberculosis (TB), which is understandable since Finland is a country with a low TB incidence.^7^ COVID‐19 infection and vaccination have recently been discussed in the literature as etiological factors behind EN.^8^ However, this study did not include cases diagnosed after December 2019; the impact of the COVID‐19 pandemic is thus not seen. We also noticed some sex differences in etiology: gastroenteritis caused by ‘Yersinia, Campylobacter and Salmonella’ was significantly more common among males compared to females. In contrast, female predominance in EN diagnosis has been hypothesized to be related to sex hormones, supported by the higher occurrence of EN in pregnant women or those using oral contraceptive pills.^3^ In our study, hormonal contraceptives and pregnancy were considered a provoking factor in 16% and 4.5% of women's EN cases, respectively.

In previous studies, the most common causative factor of EN in children has been bacterial infections and especially, β‐hemolytic Streptococcus.^4^, ^9^, ^10^ Our findings are consistent with those of other studies; however, in our study, gastroenteritis‐related EN was even more common. We also reported four cases with IBD in children. However, as reported previously,^9^, ^10^ EN was not associated with any other chronic diseases, such as Bechet's disease or malignancies, in the children in our population.

In this study, the typical clinical feature of EN was multiple nodules (95%) seen in both lower limbs (64.8%). Nevertheless, those of advanced age also presented with single nodules (14.3%). We also found that systemic symptoms, especially fever and arthralgia, were common (81%). These findings are in line with the Turkish study (*n* = 100, mean age 37 years) by Mert et al. ^6^ In their study, 99% of the cases presented with bilateral, pretibial nodules, 34% with fever, and 45% with arthralgia. An interesting finding of our study was that men presented more systemic symptoms in comparison to women and were more likely to have fever when examined. The oldest and youngest group also reported less arthralgia than the other age groups.

A strength of this study is that all patients were diagnosed in OUH, and diagnosis was based on the patient history and physical examination. However, a skin biopsy was taken in an atypical presentation of EN (i.e., if typical clinical findings, such as the acute onset of tender nodules or plaques, was lacking). Further, all the patient records were reviewed by a medical doctor and uncertain cases (miscoding/diagnoses were further confirmed to other than EN) were not included.

We admit some limitations to our study. As a retrospective study, it was not possible to standardize the diagnostic procedures. Further, not all the data, such as smoking status, were available. Because the study was conducted in hospital setting, some cases with less severe symptoms might be missing since they were treated in primary healthcare.

In conclusion, while EN usually resolves with only symptomatic treatment, it is important to exclude the underlying causes. Our study highlights the most common etiologies in Northern Europe and the differences between different age groups. The leading symptoms as well as their severity can vary drastically, which may cause the patient to attend a number of different specialist clinics. Thus, awareness of features of EN is important among all physicians.

## FUNDING INFORMATION

None.

## CONFLICT OF INTEREST STATEMENT

None declared.

## ETHICS STATEMENT

The medical director of Oulu University Hospital approved the study.

## Data Availability

Data requests can be submitted to hospital administrative authority.
